# Cerebrospinal fluid biomarkers of neuroinflammation in children with hydrocephalus and shunt malfunction

**DOI:** 10.1186/s12987-021-00237-4

**Published:** 2021-01-29

**Authors:** Carolyn A. Harris, Diego M. Morales, Rooshan Arshad, James P. McAllister, David D. Limbrick

**Affiliations:** 1 Wayne State University Dept. of Chemical Engineering and Materials Science, 6135 Woodward Avenue, Rm 1413, Detroit, MI 48202 USA; 2 Department of Neurosurgery, Washington University in St. Louis, 425 S. Euclid, St. Louis, MO 63110 USA; 3 Division of Pediatric Neurosurgery, and Department of Pediatrics, Department of Neurosurgery, Washington University in St. Louis, 425 S. Euclid, St. Louis, MO 63110 USA

**Keywords:** Neuroinflammation, Cytokines, Mmps, Hydrocephalus, Revisions, Multiplex ELISA

## Abstract

**Background:**

Approximately 30% of cerebrospinal fluid (CSF) shunt systems for hydrocephalus fail within the first year and 98% of all patients will have shunt failure in their lifetime. Obstruction remains the most common reason for shunt failure. Previous evidence suggests elevated pro-inflammatory cytokines in CSF are associated with worsening clinical outcomes in neuroinflammatory diseases. The aim of this study was to determine whether cytokines and matrix metalloproteinases (MMPs) contribute towards shunt failure in hydrocephalus.

**Methods:**

Using multiplex ELISA, this study examined shunt failure through the CSF protein concentration profiles of select pro-inflammatory and anti-inflammatory cytokines, as well as select MMPs. Interdependencies such as the past number of previous revisions, length of time implanted, patient age, and obstruction or non-obstruction revision were examined. The pro-inflammatory cytokines were IL-1β, IL-2, IL-5, IL-6, IL-8, IL-12, IL-17, TNF-α, GM-CSF, IFN-γ. The anti-inflammatory cytokines were IL-4 and IL-10, and the MMPs were MMP-2, MMP-3, MMP-7, MMP-9. Protein concentration is reported as pg/mL for each analyte.

**Results:**

Patient CSF was obtained at the time of shunt revision operation; all pediatric (< 18), totaling n = 38. IL-10, IL-6, IL-8 and MMP-7 demonstrated significantly increased concentrations in patient CSF for the non-obstructed subgroup. Etiological examination revealed IL-6 was increased in both obstructed and non-obstructed cases for PHH and congenital hydrocephalic patients, while IL-8 was higher only in PHH patients. In terms of number of past revisions, IL-10, IL-6, IL-8, MMP-7 and MMP-9 progressively increased from zero to two past revisions and then remained low for subsequent revisions. This presentation was notably absent in the obstruction subgroup. Shunts implanted for three months or less showed significantly increased concentrations of IL-6, IL-8, and MMP-7 in the obstruction subgroup. Lastly, only patients aged six months or less presented with significantly increased concentration of IL-8 and MMP-7.

**Conclusion:**

Non-obstructive cases are reported here to accompany significantly higher CSF cytokine and MMP protein levels compared to obstructive cases for IL-10, IL-6, IL-8, MMP-7 and MMP-9. A closer examination of the definition of obstruction and the role neuroinflammation plays in creating shunt obstruction in hydrocephalic patients is suggested.

## Background

Hydrocephalus predominately presents as an abnormal expansion of cerebral ventricles associated with cerebrospinal fluid (CSF) build up [1–3]. Left untreated, increased intracranial pressure and compression of brain structures can lead to neurological defects, nausea, headaches, lethargy and ultimately be fatal [3–5]. Globally, hydrocephalus has a prevalence between 11 and 175/100,000 depending on the age [6,7]. In the United States, hydrocephalus is responsible for 69,000 annual discharges, incurring a burden greater than $2.0 billion to the healthcare system [7–9]. To date, the most effective interventions for treating hydrocephalus are surgical, typically Endoscopic Third Ventriculostomy (ETV) or ventriculoperitoneal (VP) shunting, the gold standard for hydrocephalus [9,10]. Shunts are plagued by a number of complications that usually result in their failure, requiring subsequent revision and eliciting additional financial and healthcare burdens [11–13]. Proximal catheter obstruction has been reported to be the most common reason for shunt failure among pediatric hydrocephalus patients, [8,^14^,15] but it has been difficult to discern the underlying cause for shunt failure [8,^12^,^14^,^16^–20].

Cytokines are major mediators and principal signaling chemicals in immunoinflammatory processes within the human body, including the CNS. Their functions and interactions cover a wide range of physiological effects but can be generally categorized as pro-inflammatory cytokines which increase production of cytokines to increase inflammatory cascades or anti-inflammatory cytokines which inhibit release of pro-inflammatory cytokines. We hypothesized that an ever-increasing presence of pro-inflammatory cytokines would follow increased shunt revision number, assuming that obstructive masses are predominately composed of inflammatory cells [21].

MMPs are a family of proteolytic enzymes responsible for degrading and remodelling extracellular matrix proteins found in all tissues, as well as junctional proteins, growth factors, and cytokines. In cases of brain traumatic injury or neuroinflammation, increased CSF MMP expression has been observed [22]. The most abundant MMPs within the brain are MMP‐2, MMP‐3, and MMP‐9 [23] and these are implicated in neurodevelopment, neuro-regeneration, and in several pathological conditions including Alzheimer’s Disease, cancer metastasis, and viral infections [22]. Gelatinases such as MMP-2 and MMP-9 have also been reported to play an important role in angiogenesis and neurogenesis [24]. They have become increasingly implicated in modulating blood brain barrier permeability and TBI severity [25,26]. Additionally, MMP-2 and MMP-9 expression in knockout mice studies has been reported to be highly selective, rapidly inactivated and capable of modulating local chemokine and cytokine activation or inactivation [27]. MMP-3 is also reported to be involved in blood brain barrier disruption and found to have increased expression in TBI and capable of activating other MMPs including MMP-7 and MMP-9 [28]. We hypothesised that MMPs may play an important role in shunt failure in hydrocephalus.

As the mechanisms underlying shunt failure elude researchers and clinicians, it is important to thoroughly investigate the CSF and environment surrounding the shunt catheters in addition to examining the efficacy of the catheters themselves [29,30]. To that end, this study looked at the CSF protein concentration profiles of select pro- and anti-inflammatory cytokines and select MMPs from pediatric patients with various hydrocephalus etiology at the time of shunt failure. In order to gain a deeper understanding of the various interdependencies within this cohort, the role of possible factors influencing shunt failure was examined, namely: etiology, shunt revision history, length of time implanted, and age of patient. Lastly, the study compared the presentation of pro-inflammatory and anti-inflammatory cytokines as well as select MMPs between patients with obstructed and non-obstructed shunts. Identifying and understanding the relationship between the CSF and environment surrounding the shunt catheters at the molecular and cellular level is imperative towards gaining an understanding of shunt failure and facilitating better informed clinical decisions for the future.

## Materials and methods

### Ethics approval

The permission to collect CSF samples from failed shunt surgeries was approved by local ethics committees at each participating center; records of approval were sent to the coordinating center, Wayne State University (WSU), and submitted as amendments to our protocol under the Institutional Review Board (IRB). Washington University Human Research Protection Office (WU-HRPO) approval was obtained prior to beginning this study. Written informed consent was obtained from all patients or their legally authorized representative. Collection was performed in a manner consistent with the Declaration of Helsinki and represents no modification to the standard of treatment.

### Study population

CSF samples were collected from 38 patients ranging in age from neonatal to 17 years. Samples were collected from individuals with all hydrocephalus etiologies, excluding normal pressure hydrocephalus. Samples were only collected if shunt malfunction necessitated revision according to the attending neurosurgeon. Sample bins for revision history and implantation time were determined with a focus on acute timeframes while sample bins for age focused on smaller age groupings. This is in accordance with previous reporting that the highest risk of shunt failure is acute, but decreases over time and with the number of revisions [31], and the fact that hydrocephalus is more common in infants [7,9]. Data for healthy controls was retrieved from previous publications where similar multiplex ELISAs were performed using age-matched patient CSF whenever possible [29, 32–34] and added to the figures as a dotted line. All pediatric subjects underwent routine MRIs and/or CT to assist in the diagnosis of a shunt malfunction. Those found to have a possible shunt malfunction were then treated with or without surgery at the discretion of the neurosurgeon. As previously described [35] CSF was collected at the time of shunt surgery and transported on ice to the Washington University Neonatal CSF Repository. Samples were then centrifuged (2500 rpm for 6 min) at room temperature, and the supernatant was aliquoted and stored in 1.5 ml polypropylene microcentrifuge tubes at − 80 °C until experimental analysis.

### Data collection and multiplex assay

Multiplex assays were run by the Bursky Center for Human Immunology & Immunotherapy Programs (CHiiPs) at Washington University School of Medicine according to the manufacturer’s instructions. Frozen supernatant CSF was slowly thawed and then analyzed in duplicate with multiplex kits (Thermofisher Scientific, Waltham, MA) for the following pro-inflammatory cytokines: IL-1β, IL-2, IL-5, IL-6, IL-8, IL-12, IL-17, TNF-α, GM-CSF, IFN-γ; and anti-inflammatory cytokines: IL-4 and IL-10; as well as selected MMPs: MMP-2, MMP-3, MMP-9, MMP-7 (catalog number EPXP130-10100-901, EPX03A-10829-901, PPX-01-MMP7). Briefly, magnetic beads were added across all the wells on the plate; CSF samples and standards were then added in duplicate. Following washing steps, the detection antibody was added followed by streptavidin incubation. Beads were then resuspended with reading buffer and data were acquired on a Luminex system. The concentration of each antigen was calculated by plotting the expected concentration of the standards against the multiplex fluorescent immunoassay generated by each standard. A 4-parameter logistic regression was used for the best fit curve. Protein concentration is reported as pg/mL for each analyte.

### Data presentation and statistical analysis

All data presentation was performed using Graphpad Prism version 8.4.0 in MacOS. Mean difference between groups with a 95% confidence interval is shown. Two-way ANOVA comparisons were conducted to determine interdependency of past revision number, length of implantation time, etiology and age, on cytokine and MMP protein concentration in CSF, assuming unequal variances. A predetermined significance level of 0.05 was used in all statistical tests.

## Results

Comparisons were made for protein concentration in CSF for the following pro-inflammatory cytokines: IL-1β, IL-2, IL-5, IL-6, IL-8, IL-12, IL-17, TNF-α, GM-CSF, IFN-γ; and anti-inflammatory cytokines: IL-4 and IL-10; as well as select MMPs: MMP-2, MMP-3, MMP-7, MMP-9. Of these aforementioned cytokines and MMPs, those that did not show any significant differences between the various groupings were not reported in Figs. 3, 4, 5, 6.

### Patient characteristics

Table 1 includes a distribution of patient etiology. A total of 38 subjects were enrolled and CSF samples were collected over the span of 46 months (May 2015 to March 2019). Twenty of the subjects were male (52.63%) and 18 female (47.36%); 31 were Caucasian, 6 African American, and one unreported. Their age ranged from neonates up to 17 years, with 12 CSF samples collected prior to age one, nine of toddler age (ages 1–3), and three preschoolers (ages 4–5). The mid-childhood children (ages 6–12) and the teenagers (ages 13–17) had seven individuals in each age group. As shown in Table 1, 15 had PHH etiology while myelomeningocele (6) and aqueductal stenosis (3) were also prominent.

**Table 1:** Distribution of etiologies and contained samples per etiology

Generalized etiology of hydrocephalus	Specific etiology of hydrocephalus	Patient count	Avg. patient age (years)	Males:females
Communicating	Post-hemorrhagic Hydrocephalus	15	4.54	6:9
Communicating	Infection	2	13.53	2:0
Communicating	Porencephalic cyst	1	0.09	0:1
Communicating	Tumor	1	0.41	0:1
Non-communicating	Myelomeningocele	6	3.51	2:4
Non-communicating	Aqueductal Stenosis	3	4.88	3:0
Non-communicating	Congenital Hydrocephalus	3	15.3	2:1
Non-communicating	Chiari 1	1	17.28	0:1
Non-communicating	Communicating Hydrocephalus	1	2.99	1:0
Non-communicating	Dandy-Walker Malformation	1	13.08	0:1
Non-communicating	Obstructive	1	4.72	1:0
Traumatic brain injury	Traumatic brain injury	3	16.74	3:0
	*Averages*		*8.089166667*	
	*Totals*	*38*		*20:18*

**Fig. 1:**
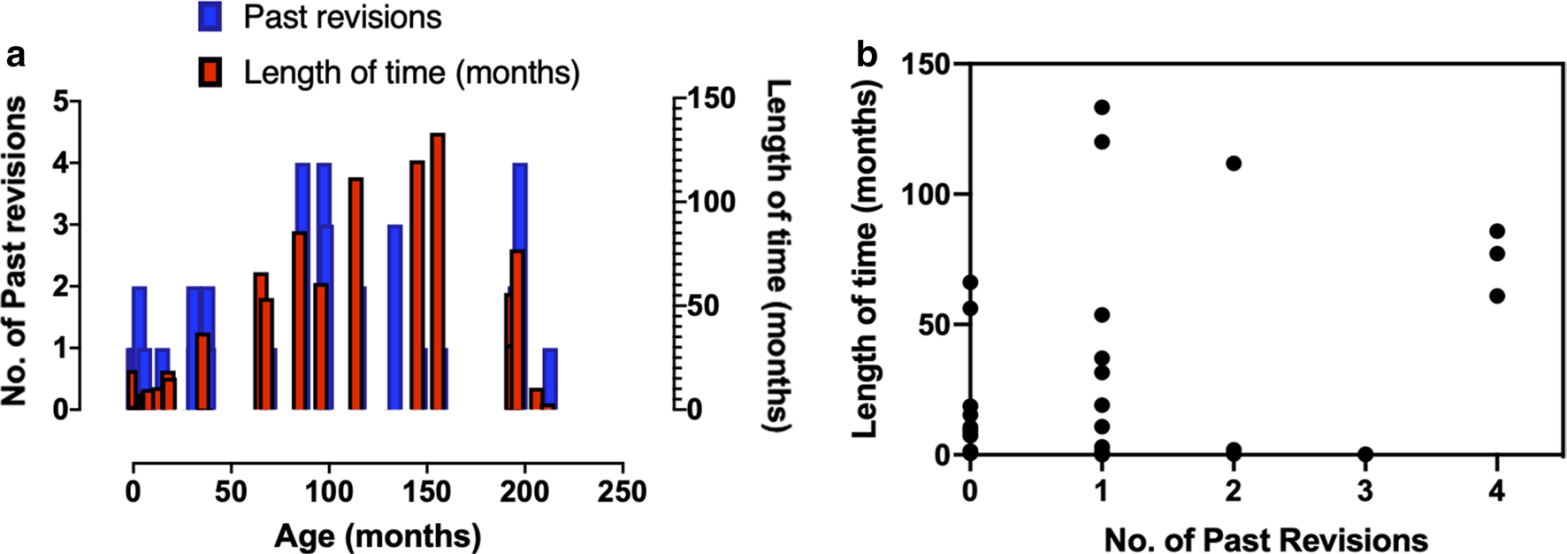
Distribution of samples related to **a** the number of past revisions, age and length of time implanted, and **b** length of time implanted, and number of past shunt revisions

Figure 1a shows the distribution of samples in relation to length of time implanted, age and number of past revisions. Figure 1a shows that shunt failure when distributed according to number of past revisions and length of time both lack a clear clustering according to age. However, a closer look at the data is shown in Fig. 1b which demonstrates the distribution of times to shunt revision (length of time implanted) for samples in relation to number of past revisions. Figure 1b shows that the majority of revisions were performed in the first 50 months for individuals with 1–3 past revisions. Individuals with 0 past revisions (n = 12) had revisions performed less than 67 months, majority (n = 10) of which were performed within 20 months. Individuals with 1 past revision (n = 13) presented with the most variance, with 2 individuals exceeding time to shunt revision by 120 months while the remaining majority (n = 11) had revisions performed in less than 55 months. Individuals with 2 past revisions (n = 4) presented within 2 months of time to shunt revision, with one exception of 111 months. Individuals with 3 past revisions (n = 2) presented with the shortest time to shunt revision of roughly 4 days for both. All revisions for individuals with 4 past revisions (n = 3) were performed between 60 to 86 months.

### Obstruction vs. non-obstructed

**Fig. 2:**
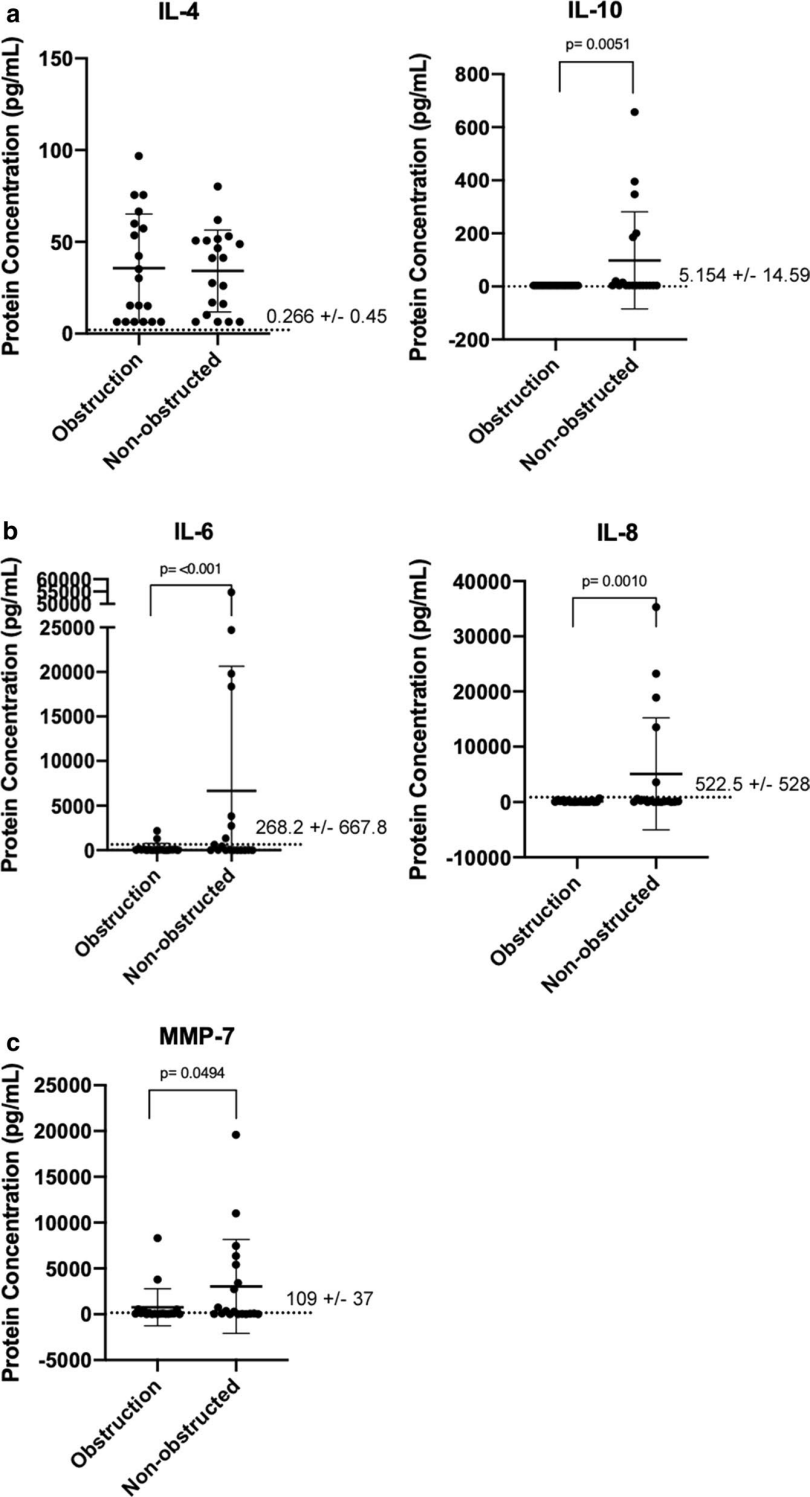
CSF (Cerebrospinal fluid) cytokine and MMP protein concentrations between obstructive cases or non-obstructive cases (n = 19 per group). Analytes showed include **a** anti-inflammatory cytokines, **b** pro-inflammatory cytokines and **c** MMPs (Matrix Metalloproteinase). Mean with standard deviation is shown in error bars

All samples within the dataset were divided according to clinical determination of whether the shunt catheter was being revised due to obstruction (n = 19; 17 proximal and 2 distal) or for non-obstruction reasons (n = 19; over drainage, loculations, infection, etc.). As Fig. 2a–c show, none but IL-10 (p = 0.0051), IL-6 (p =  < 0.0001), IL-8 (p =  < 0.0010) and MMP-7 (p = 0.0494) demonstrated significantly increased concentration in patient CSF in the non-obstructed subgroup compared to the obstructed subgroup. This increase in CSF protein concentration was primarily observed in female individuals with PHH whose shunt was revised due to loculation. This demonstrated that the variance in the cytokine and MMP protein concentration in hydrocephalus patients required further analysis. Therefore, the data were examined for the influence of the following factors on protein concentration: past number of previous revisions, length of time implanted, age and etiology in each category subdivided into reason for revision.

### Etiology

**Fig. 3:**
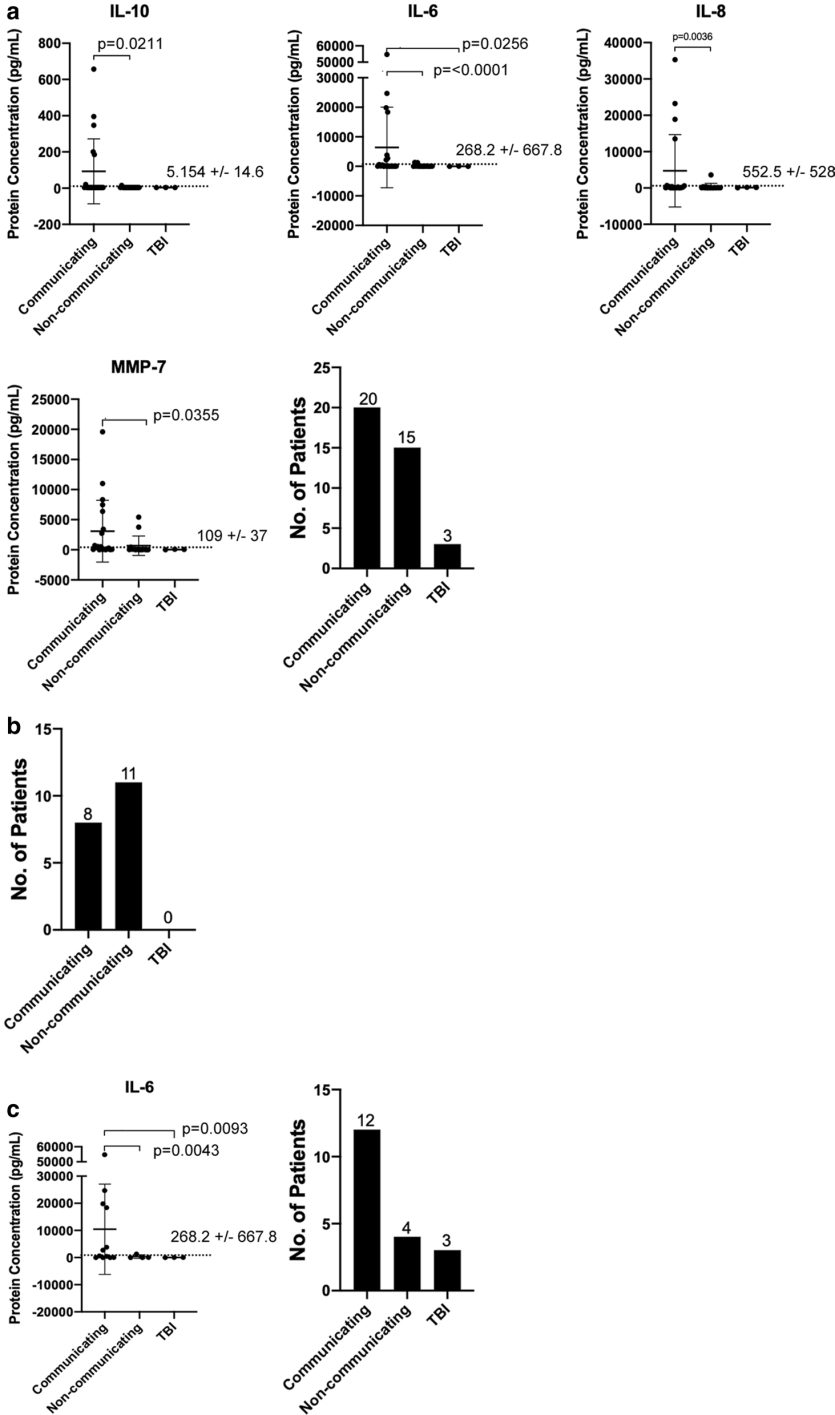
Protein concentrations of select cytokines and MMPs after subdivision into communicating HCP, non-communicating HCP and traumatic brain injury (TBI) cases for each of the following groups: **a** unparsed data, **b** obstructed cases, **c** non-obstructed cases. Mean with standard deviation is shown in error bars

As Fig. 3a shows, IL-10, IL-8 and MMP-7 demonstrated significantly increased concentrations in patient CSF for communicating cases compared to non-communicating cases, while only IL-6 is significantly higher than both non-communicating and TBI cases. Further examination of the etiologies within the cohort, as included in Additional file 1: Figure S1A: aqueductal stenosis (n = 3), congenital hydrocephalus (n = 3), infection (n = 2), myelomeningocele (n = 6), traumatic brain injury (TBI) (n = 3) and individuals with PHH being the most prevalent (n = 15). IL-6 was significantly increased in patients with PHH compared to those with aqueductal stenosis (p = 0.0295), myelomeningocele (p = 0.0002), and TBI (p = 0.0282). IL-8 was significantly higher only in PHH patients when compared to patients with myelomeningocele (p = 0.0144).

Subsequently, data were parsed according to the reason for revision: obstruction or non-obstruction (n = 19 for both) for Fig. 3b and c, respectively. In obstruction cases, no cytokines or MMPs are significantly changed within the general etiologies. Interestingly, only IL-6 was determined to be significantly higher in communicating cases and showed a similar profile as seen in Fig. 3a. In contrast, Additional file 1: Figure S1B (for obstruction cases) shows IL-6 as the sole cytokine with significantly higher expression in individuals with congenital hydrocephalus (p = 0.0126, p = 0.0003) and PHH (p = 0.0018) compared to other specific etiologies. For non-obstructed cases as Fig. 3c shows, it is to be noted IL-6 remains the only significantly higher expressed cytokine in individuals with PHH compared to TBI in the non-obstructed subgroup (p = 0.0169).

### Revision history

**Fig. 4:**
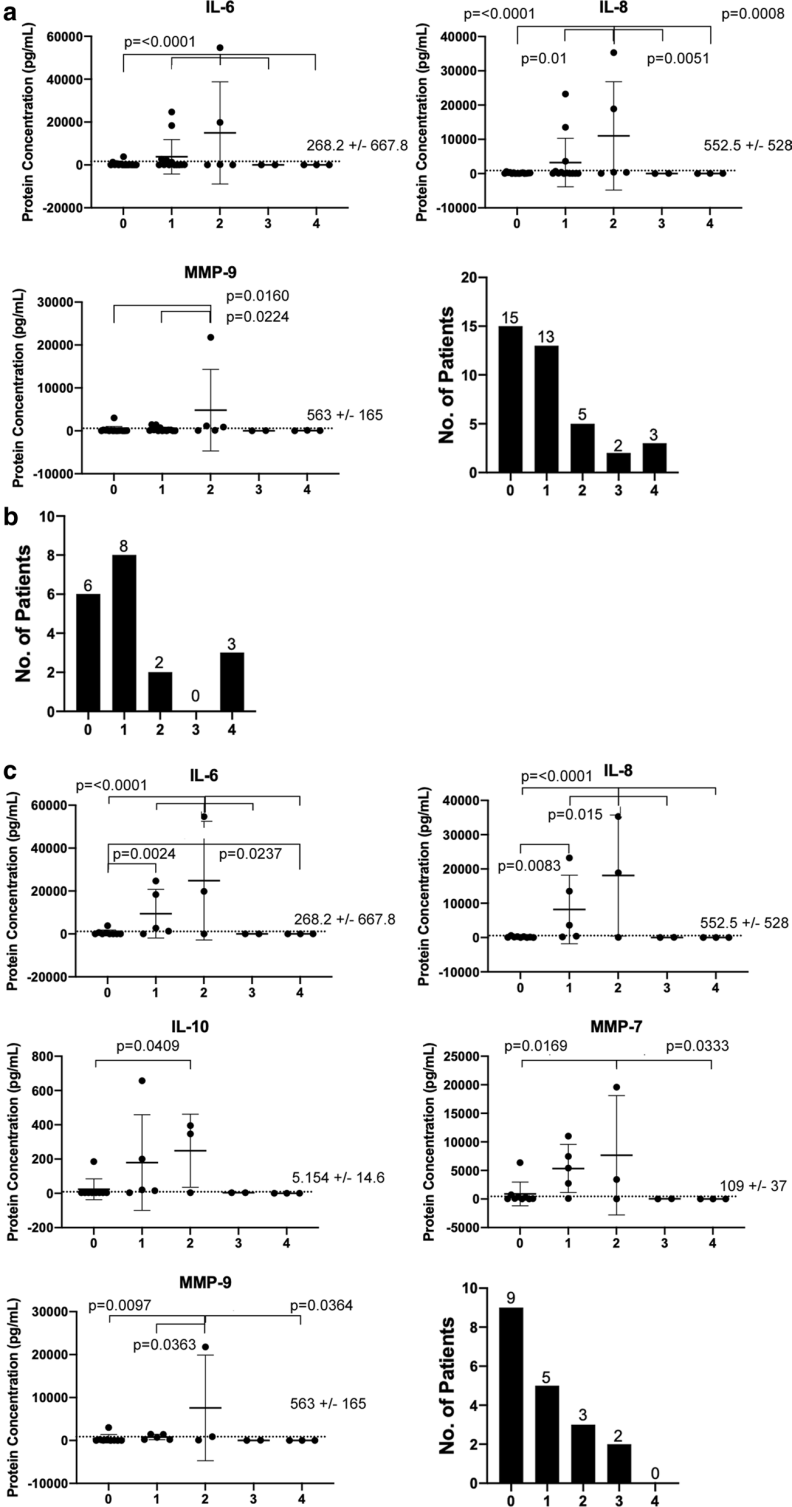
Protein concentration values of CSF (Cerebrospinal fluid) cytokines and MMPs (Matrix Metalloproteinase) after samples were subdivided by number of past revisions, as well as sample count per revisions are reported: **a** unparsed data, then **b** obstructed vs. **c** non-obstructed cases. Mean with standard deviation is shown in error bars

Data were parsed according to revision history to determine if a correlation exists between the patient’s revision history (quantified as the number of past revisions) and select neuroinflammatory cytokines and MMPs. Figure 4a includes all samples within the cohort without regard to reason for revision, while Fig. 4b includes protein concentration within the obstruction subgroup and Fig. 4c includes protein concentration within the non-obstruction subgroup. Figure 4a shows, in the case of all-inclusive data, only IL-6 (p =  < 0.0001) and IL-8 (p =  < 0.0051) are observed to hold significantly higher expression at the point of two previous revisions compared to the preceding and subsequent number of past revisions, but also a sharp drop in CSF protein concentration is observed for individuals with three previous revisions and onward. Additional file 2: Figure S2A and Additional file 3: Figure S3 further demonstrate this for all the remaining cytokines. Similarly, MMP-9 (p =  < 0.0224) is observed to hold significantly higher expression from zero to two previous revisions but not for the next number of past revisions. Additional file 6: Figure S6 further demonstrates this for all the remaining MMPs.

Interestingly, none of the cytokines or MMPs demonstrate significantly variant expression in terms of interdependency on the number of past revisions in the obstructed subgroup. This pattern is absent in all other cytokines and MMPs for obstruction cases (Additional file 2: Figure S2B, Additional file 4: Figure S4, Additional file 6: Figure S6). In contrast, within the non-obstructed subgroup (Fig. 4c), IL-10, IL-6 IL-8, MMP-7 and MMP-9 show the same concentration profiles between the different numbers of past revisions. IL-10 (p = 0.0409) was observed to be significantly increased at two past revisions compared to zero past revisions, but within the error rate for one past revisions. IL-6 (p =  < 0.03) and IL-8 (p =  < 0.01) were determined to have similar protein concentration profiles across the revision time points. IL-6 and IL-8 protein concentrations progressively and significantly increase from zero to two previous revisions and then a sharp drop is observed for individuals with three and four previous revisions. MMP-7 (p =  < 0.0333) was shown to be significantly increased at two past revisions compared to zero past revisions, but within the error rate for one past revisions, with a sharp decline following the point of two past revisions. Additional file 2: Figure S2C and Additional file 5: Figure S5 further demonstrate this for all the remaining cytokines. MMP-9 (p =  < 0.0364) was observed to have a similar protein concentration profile as IL-6 and IL-8, where protein concentration progressively and significantly increases from zero to two previous revisions and then a sharp drop is observed past that point. Additional file 6: Figure S6 further demonstrates this for all the remaining MMPs.

### Implantation time

**Fig. 5:**
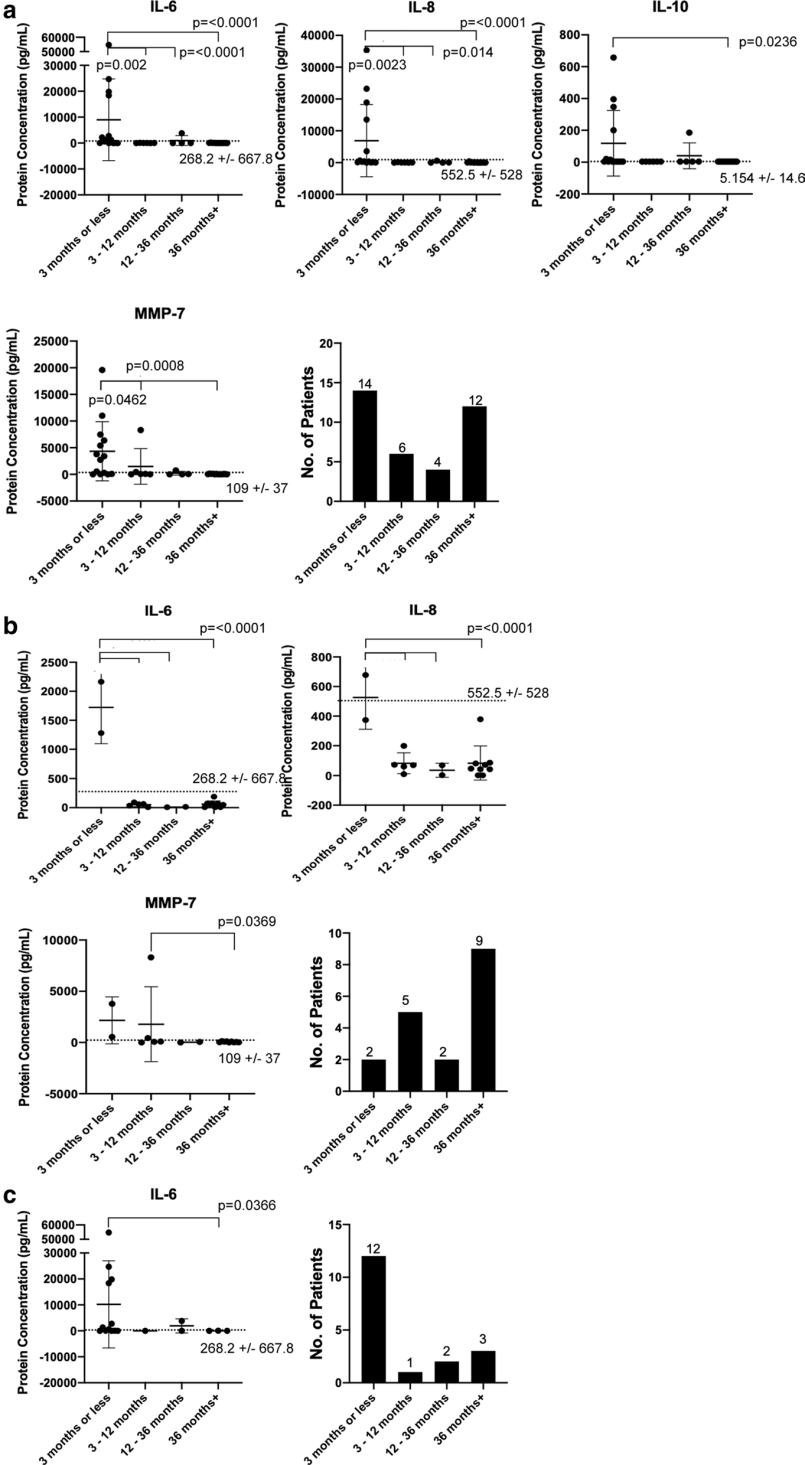
Frequency of patients in the biobank and CSF (Cerebrospinal fluid) cytokines and MMP (Matrix Metalloproteinase) protein concentrations based on length of implantation are reported: **a** unparsed data, then **b** obstructed vs. **c** non-obstructed cases. Mean with standard deviation is shown in error bars

Next, protein concentration of neuroinflammatory cytokines and MMPs was examined to determine whether an interdependency on the implantation time existed. In Fig. 5a, within the unparsed data IL-10, IL-6, IL-8, and MMP-7 are observed to have significant fluctuations in protein concentration when implantation times are compared. IL-10 (p = 0.0236) demonstrated significantly more protein concentration within the 3 months or less period compared to 36 months + . IL-6 (p =  < 0.002) and IL-8 (p =  < 0.014) were observed to have significantly higher protein concentration in the 3 months or less period compared to all other time points. Lastly, MMP-7 (p =  < 0.0462) was significantly higher in the 3 months or less period compared to 12 months and after period, but within standard deviation for the 3–12 months period.

Next, the data were parsed with regard to reason for revision, i.e., obstruction or non-obstruction. As Fig. 5b shows, within the obstructed subgroup IL-6 (p =  < 0.0001) and IL-8 (p =  < 0.0001) were observed to have the same significantly higher protein concentration profile as the unparsed data, i.e., in the 3 months or less period compared to all other time points. MMP-7 (p = 0.0369) was observed to be relatively the same for the first year, and then significantly decreased by the 36 months + time period. In the non-obstructed subgroup, Fig. 5c shows only IL-6 (p = 0.0366) was observed to be significantly higher in the 3 months or less period and then remained low for the following periods.

### Age

**Fig. 6:**
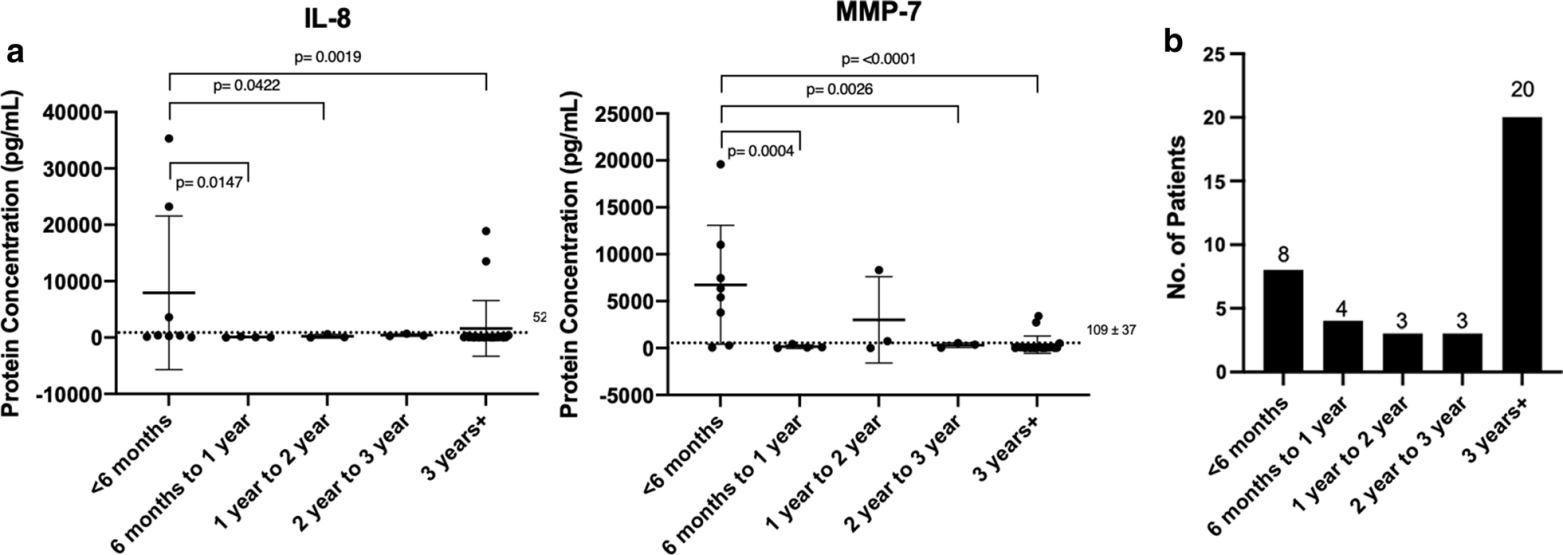
Frequency of **a** CSF (Cerebrospinal fluid) cytokines and MMP (Matrix Metalloproteinase) protein concentrations in terms of age and **b** number of patients in the biobank are reported. Mean with standard deviation is shown in error bars

Lastly, determining the dependency of age on CSF cytokines and MMP protein concentration, the ages were divided up into 6-month periods for the 1st year, then yearlong periods leading up to 3 years, while ages 3 years and more were grouped together. In the unparsed data, Fig. 6a, IL-8 and MMP-7 stand out. IL-8 (p =  < 0.0422) is significantly higher in the first 6 months in our dataset compared to the rest of the age groups, with the exception of 2–3 years of age. MMP-7 (p =  < 0.00261) was observed to be significantly decreased in all ages in our data set, except for less than 6 months and 1–2 years of age.

## Discussion

Of the tested cytokines and MMPs, protein concentrations in CSF were observed to be significantly increased for the following: pro-inflammatory cytokines IL-6 and IL-8, anti-inflammatory cytokine IL-10, MMP-7 and MMP-9.Our results suggest CSF cytokine expression and MMP activation is generally less or within error for individuals undergoing shunt revision due to symptoms of obstruction compared to shunts that failed for non-obstructed reasons (Figs. 2, 3, 4, 5, 6, Additional file 3: Figure S3, Additional file 4: Figure S4, Additional file 5: Figure S5, Additional file 6: Figure S6). However, some major cytokines and select MMPs stand out consistently. Those proteins with a significant increase in non-obstructed cases were IL-10, IL-6, IL-8, and MMP-7. Low sample size may preclude this as a predictor, however. Inclusion of referenced healthy controls as baseline shows the protein concentration levels for almost all the cytokines and MMPs shown in Figs. 3, 4, 5, 6, are either elevated or at baseline in all the dependencies examined.

Sorting by etiology reveals the aforementioned major cytokines to be elevated for communicating hydrocephalus and for non-obstructed cases. A deeper look at etiology shows IL-6 to be significantly increased in both obstructed and non-obstructed individuals, primarily for PHH and congenital hydrocephalic patients. Whereas IL-8 significantly increased in PHH patients only when the data is unparsed, but not when parsed between obstructed or non-obstructed cases. The lack of significant change in other cytokines and MMPs in other etiologies, even after subdividing data according to obstructed or non-obstructed cases, suggests a high degree of variance exists in the patient population. This variance may be related to how they respond towards recovering, compensating or aggravating hydrocephalus pathophysiology [6,^9^,12].

Parsing the data according to past number of revisions reveals that none of the analytes are significantly increased in obstruction cases. However, in the non-obstructed cases an interesting trend persists. Protein concentration levels for IL-10, IL-6, IL-8, MMP-7 and MMP-9 significantly and progressively increase from zero to two past revisions but then sharply drop for patients with three past revisions. It should be noted those patients with three past revisions only had their shunts implanted for less than four days and required revision. Whereas the four patients who came in with four or more past revisions had theirs left implanted for more than five years and needed revision after said extended length of time due to obstruction in four of seven cases (Fig. 2b). One reason for this sharp decline in cytokine and MMP presentation may be due to the low sample count, or due to patient CSF being collected at the time of shunt failure/revision surgery. In this latter case, the elusive event(s) responsible for CSF shunt system failure could have already come to pass prior to, or as the patient’s symptoms developed, or even digressed by the time of CSF collection. Additionally, small sample sizes per bin limit our ability to detect significant relationships in the obstruction cases as none of the cytokines significantly changed between the different revision attempts. However, as obstruction remains the most common reported reason for shunt failure, a more comprehensive, larger sampled review of patient CSF would aid towards understanding the neuroinflammatory mechanisms driving shunt obstruction [15].

As the role of immune mediators and MMPs in the hydrocephalic brain is currently in the stage of being profiled, the lack of outright, directional neuroinflammatory response over multiple cytokines and MMPs brings into question the extent to which neuroinflammation is a driving force behind shunt obstruction and therefore, consequent shunt failure. A longitudinal study with a large patient cohort, comprehensive in nature would be able to discern why protein concentration levels for major cytokines such as IL-10, IL-6 and IL-8, as well as MMP-7 and MMP-9 progressively and consistently rise for individuals with two past revisions but then drop to healthy control/baseline levels by the third revision. Overall, IL-10, IL-6 and IL-8 consistently standout and provide inspiration for future therapeutic control of neuroinflammation towards mitigating poor clinical outcomes. This study also brings to light the necessity to examine the failed shunt catheters, as clinical signs and symptoms of obstruction may not always equate to complete physical shunt catheter obstruction.

Investigations into the dependency of the implantation time or simply, time to shunt revision, suggests 3 months or less as the marked time with significantly increased concentration of IL-6, IL-8, and MMP-7. In contrast to the past revisions, the obstruction subgroup presented with significantly increased for implantation time compared to the non-obstruction subgroup. Similar to the past revisions, the analytes which standout are IL-6, IL-8, and MMP-7. These analytes were also significantly increased for individuals aged six months or less. This age group was identified as the most vulnerable age with significantly increased concentration of IL-6, IL-8, and MMP-7. Intuitively, both the shortest length of implantation time and the youngest age group have the highest protein concentration presentation of neuroinflammatory cytokines and of the yet understudied MMP-7. The risk for shunt failure has been previously reported to be the highest in the early stages of hydrocephalus and the risk for shunt failure decreases over time and over number of revised shunts. Our study consolidates these reports through increased presentation in the three months or less of implantation time, and through the sharp decline of major neuroinflammatory mediators by three past revisions point [31]. The CSF cytokines evaluated in this paper, and their subsequent effects on activating immune responses in vivo*,* are measurable and could be used for quantifying the degree of hydrocephalus severity in clinics as a useful biomarker and potential predictor for clinical decision making. As infants are arguably the most vulnerable group suffering from hydrocephalus [7,9], the presence of neuroinflammation and dysregulated MMPs could be contributing to loss of tissue integrity, worsening of clinical symptoms and signs, and consequently, impeding normal neurological development [3,4].

We report IL-6, IL-8 and IL-10 to be the major inflammatory cytokines of interest as they consistently stand out by being significantly elevated. This is in-line with previously published work on neurologic damage in hydrocephalus [36]. IL-6 is a multi-functional, major cytokine involved in development, neurogenesis, brain injury and neurodegeneration, in addition to mounting an immune response and inducing astrogliosis, astrocyte proliferation, and angiogenesis for recovery in the central nervous system [37,38]. While IL-8 behaves in many similar ways as IL-6, it retains a longer half-life [39–41]. IL-10 as a anti-inflammatory, neuro-protective cytokine is recognized to promote neuronal and glial cell survival and by inhibiting pro-inflammatory cytokine production to decrease neuroinflammation [42,43]. Although, benefit is wrought from these pro-inflammatory mediators in instances of viral or bacterial infection [33,41], clinical outcomes such as revision history and time to revision for hydrocephalic patients seem to be at the very least influenced by these analytes and can therefore be mitigated by them. It has been purported that IL-8 and IL-10 have a dichotomy towards brain volume, as well as grey and white matter formation with their interaction significantly modulating neuroinflammatory responses [44]. In our study, CSF IL-8 and IL-10 protein concentrations are significantly increased in almost all of the interdependencies examined, with the exception of age. Future investigations such as the addition of supplemental MMP-9 and/or IL-10, or the addition of pharmaceuticals counteracting IL-6 and IL-8, could help in understanding if the severity of hydrocephalus can be reversed, paused or mitigated.

We show that in addition to pro- and anti-neuroinflammatory cytokines, MMPs also have heterogenous presentation in hydrocephalic CSF. MMPs are known to digest extracellular matrix proteins, but little is known about their neuroprotective or neuroinflammatory role in hydrocephalus. Similar to cytokines, MMPs in the brain play a role in neurogenesis, central nervous system survival and development, neuronal myelination, integrity of the blood–brain barrier, and neuroinflammation inhibition [45–48]. Some studies have shown increased MMP-9 disrupts the blood–brain barrier and could even be responsible for spontaneous hydrocephalus [49,50], while others have shown, in infants with PHH, MMP-9 can help them overcome symptoms of hydrocephalus [51]. Therefore, the interplay of IL-6, IL-8, IL-10, MMP-7 and MMP-9 is worth future investigations to improve our understanding of the molecular and cellular events driving hydrocephalus.

### Limitations

A likely potential side effect of CSF being collected at one center is the high occurrence of PHH in our dataset. This study also suffers from small sample size, which becomes apparent when data is parsed and grouped by obstruction or non-obstruction/other reasons for revision surgery over each of the interdependencies evaluated. This study also heavily relies on the surgeon’s observations at surgery—obstruction or non-obstruction. It does not evaluate the nature of the obstruction, the degree of the obstruction nor its localization. Future work with larger sample sizes and a closer examination of the nature of obstruction on the shunt catheter would definitively add towards achieving better understanding of hydrocephalus.

Another limitation of this paper is due to the methods adopted to capture patient CSF. Many cases of shunt failure are captured emergently or during off-peak hours, meaning CSF captured by research personnel or clinicians incurs an inherent latency in capture speed. Additionally, to date there is no comprehensive hydrocephalus-focused CSF collection project focused on shunting and shunt failure and their relationship with neuroinflammation. One way of circumventing this would be by undertaking a large cohort comprehensive approach which involves siphoning off patient CSF at regular intervals or CSF drained transiently for specific amounts, to be stored appropriately and right away for future analysis. However, this approach presents itself with ethical, logistical and compliance challenges which would be difficult to overcome even within a controlled, clinical setting.

## Conclusion

In summary, this study is the first to examine the protein-level expression profile of MMPs in concert with select pro- and anti-inflammatory cytokines implicated in neuroinflammation within a hydrocephalus patient population at times of shunt revision. IL-10, IL-6, IL-8, MMP-7 and MMP-9 were observed to be significantly elevated compared to other cytokines and MMPs, and several magnitudes higher than their normal, non-hydrocephalic CSF counterparts. Patients with PHH had the highest CSF protein concentrations for the analytes listed above. Patients with ages of six months or less and those patients whose shunts were implanted for 3 months or less were identified as the most vulnerable groups. Under non-obstructive conditions of hydrocephalus, patient CSF showed significantly increased values of neuro-inflammatory cytokines. Interestingly, individuals with non-obstructive PHH reflected the largest increase in multiple cytokines and MMP protein levels among hydrocephalic patients.

The work presented here yields preliminary data to guide the direction of future experiments by shedding light on the expression profile of select cytokines and MMPs in patient CSF immediately prior to revision surgeries. A more comprehensive review of CSF obtained from hydrocephalus patients is very much required to give researchers and clinicians a satisfactory looking glass into the inner workings of the diseased hydrocephalus brain, as unveiling the mystery of hydrocephalus will undoubtedly give us a better understanding of means to mitigate the disorder but also elucidate inner workings of the human brain.

## Supplementary Information

**Additional file 1: Figure S1.** Following subdivision by specific etiology, protein concentration values of select cytokines and MMPs (Matrix Metalloproteinase) and sample count per etiology of each of the following groups are reported: (A) unparsed data, (B) then obstructed vs. (C) non-obstructed cases. Mean with standard deviation is shown in error bars.

**Additional file 2: Figure S2.** Protein concentration values of anti-inflammatory cytokines in the purview of past revisions: (A) unparsed data, then (B) obstructed vs. (C) non-obstructed cases. Mean with standard deviation is shown in error bars.

**Additional file 3: Figure S3.** Protein concentration values of select pro-inflammatory cytokines in terms of past revisions: unparsed data. Mean with standard deviation is shown in error bars.

**Additional file 4: Figure S4.** Protein concentration values of select pro-inflammatory cytokines in terms of past revisions: obstructed cases. Mean with standard deviation is shown in error bars.

**Additional file 5: Figure S5.** Protein concentration values of select pro-inflammatory cytokines in terms of past revisions: non-obstructed cases. Mean with standard deviation is shown in error bars.

**Additional file 6: Figure S6.** Protein concentration values of select MMPs (Matrix Metalloproteinase) in terms of past revisions: (A) unparsed data, then (B) obstructed vs. (C) non-obstructed cases. Mean with standard deviation is shown in error bars.

## Abbreviations

CSF: Cerebrospinal fluid
MMP: Matrix metalloproteinases
ETV: Endoscopic third ventriculostomy
PHH: Post-hemorrhagic hydrocephalus
IL: Interleukin
TNF-α: Tumor necrosis factor alpha
GM-CSF: Granulocyte–macrophage colony-stimulating factor
IFN-γ: Interferon gamma
VP: Ventriculoperitoneal
HCP: Hydrocephalus
