# Supplementary figures and images for: Cerebrospinal fluid biomarkers of neuroinflammation in children with hydrocephalus and shunt malfunction
Source: Fluids Barriers CNS. 2021 Jan 29;18:4. doi: 10.1186/s12987-021-00237-4 (PMC7845119; doi:10.1186/s12987-021-00237-4)

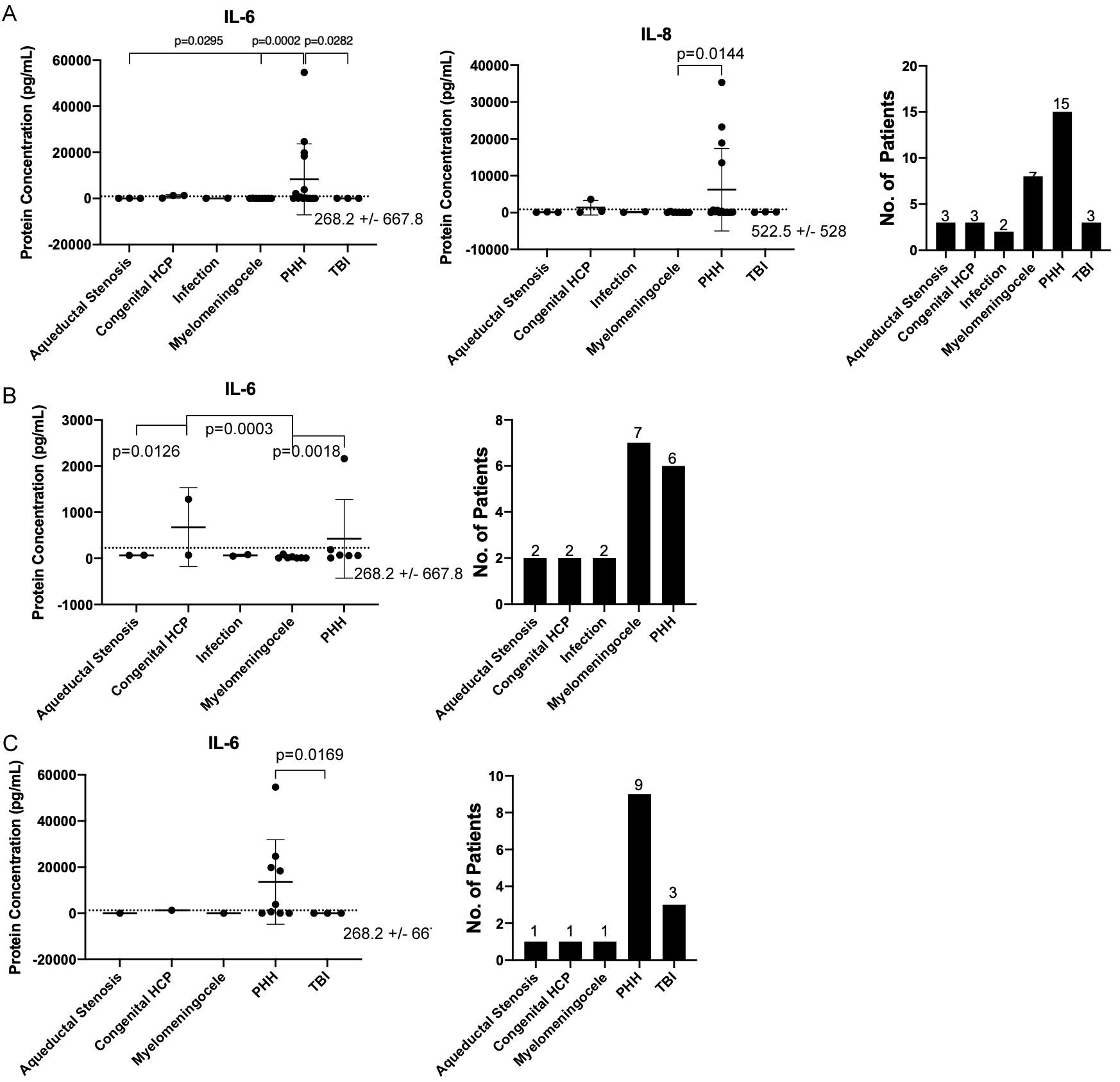

Supplement: Supplementary file 1 — Additional file 1: Figure S1. Following subdivision by specific etiology, protein concentration values of select cytokines and MMPs (Matrix Metalloproteinase) and sample count per etiology of each of the following groups are reported: (A) unparsed data, (B) then obstructed vs. (C) non-obstructed cases. Mean with standard deviation is shown in error bars. [file 12987_2021_237_MOESM1_ESM.png]

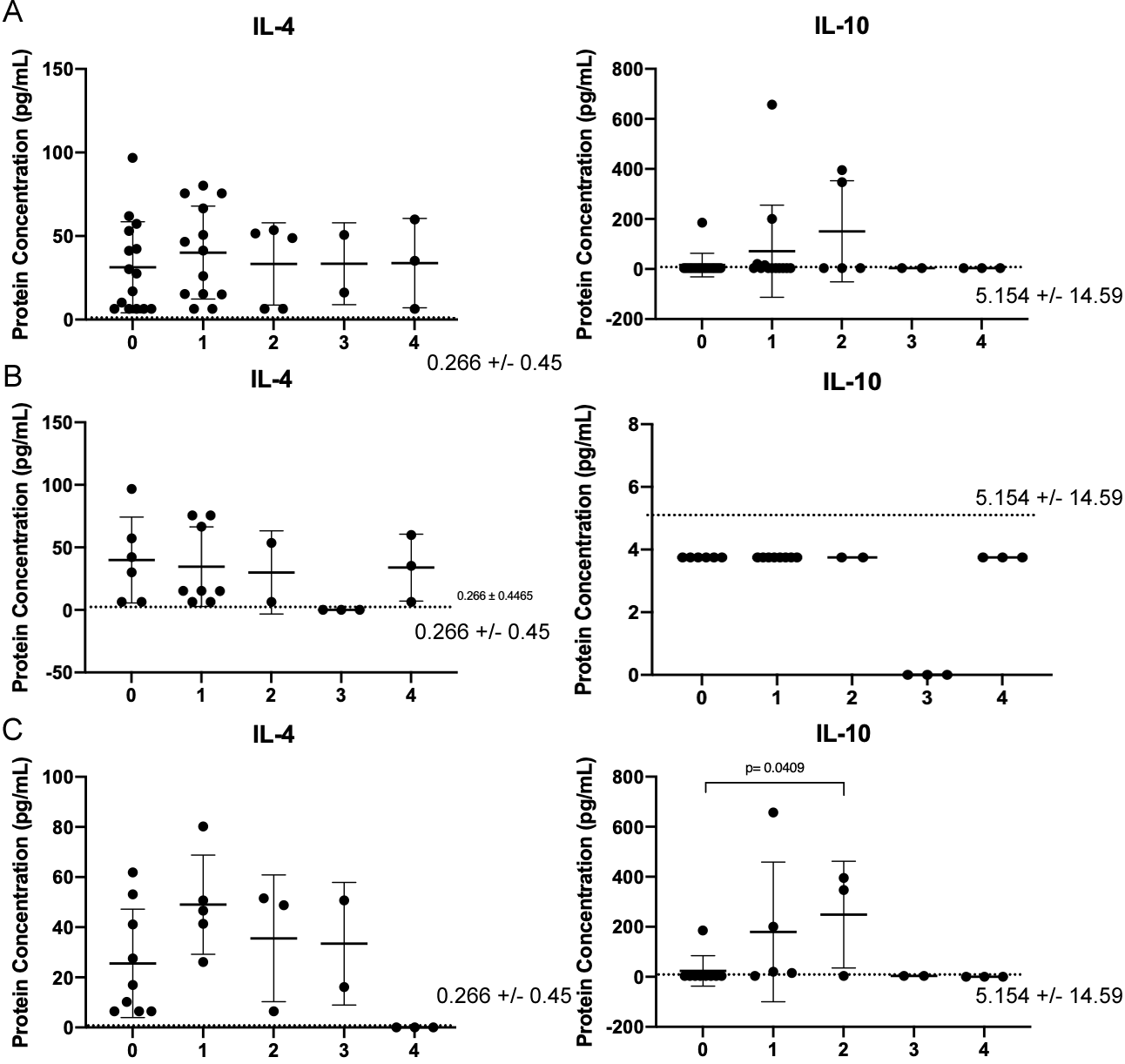

Supplement: Supplementary file 2 — Additional file 2: Figure S2. Protein concentration values of anti-inflammatory cytokines in the purview of past revisions: (A) unparsed data, then (B) obstructed vs. (C) non-obstructed cases. Mean with standard deviation is shown in error bars. [file 12987_2021_237_MOESM2_ESM.png]

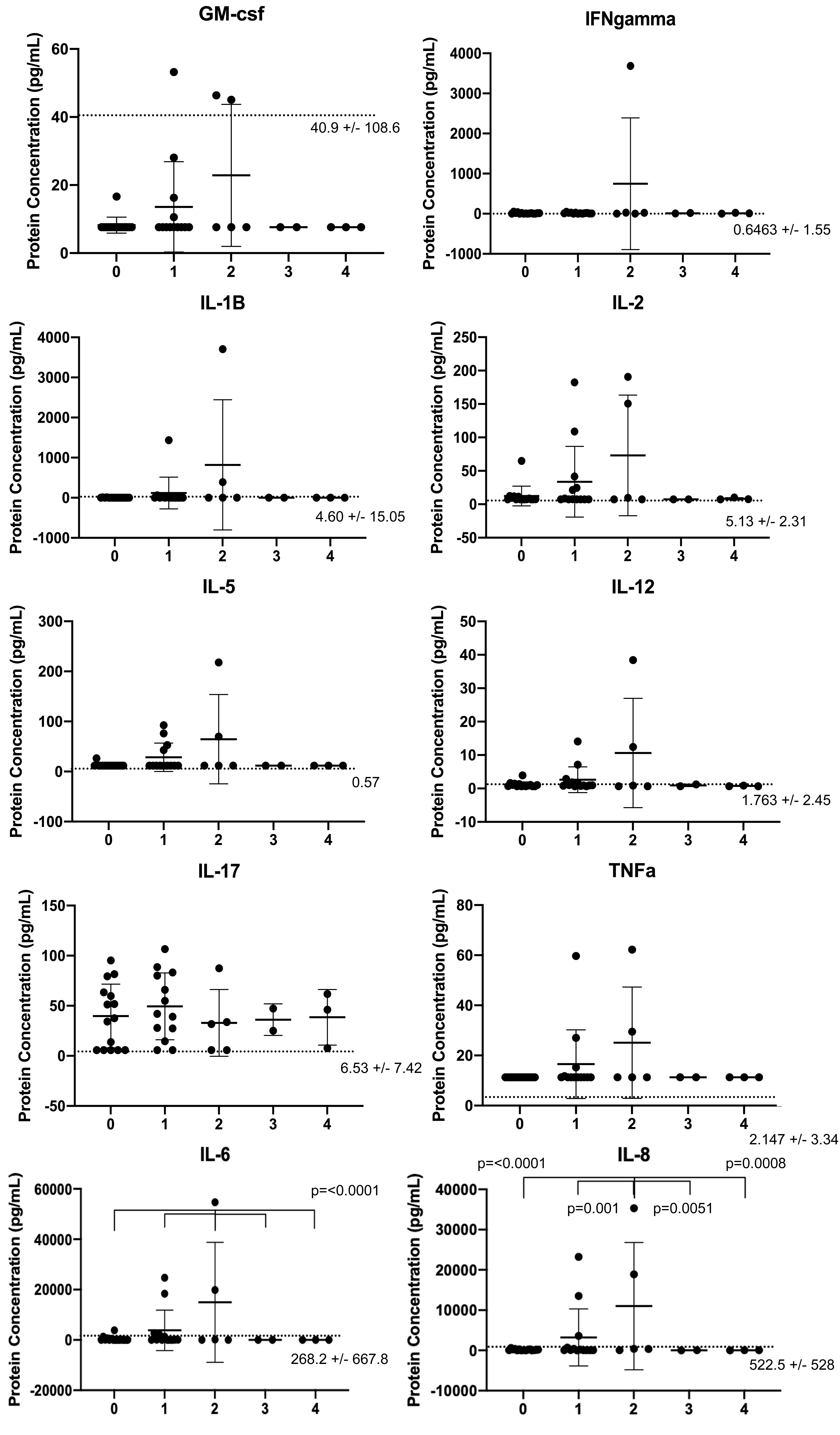

Supplement: Supplementary file 3 — Additional file 3: Figure S3. Protein concentration values of select pro-inflammatory cytokines in terms of past revisions: unparsed data. Mean with standard deviation is shown in error bars. [file 12987_2021_237_MOESM3_ESM.png]

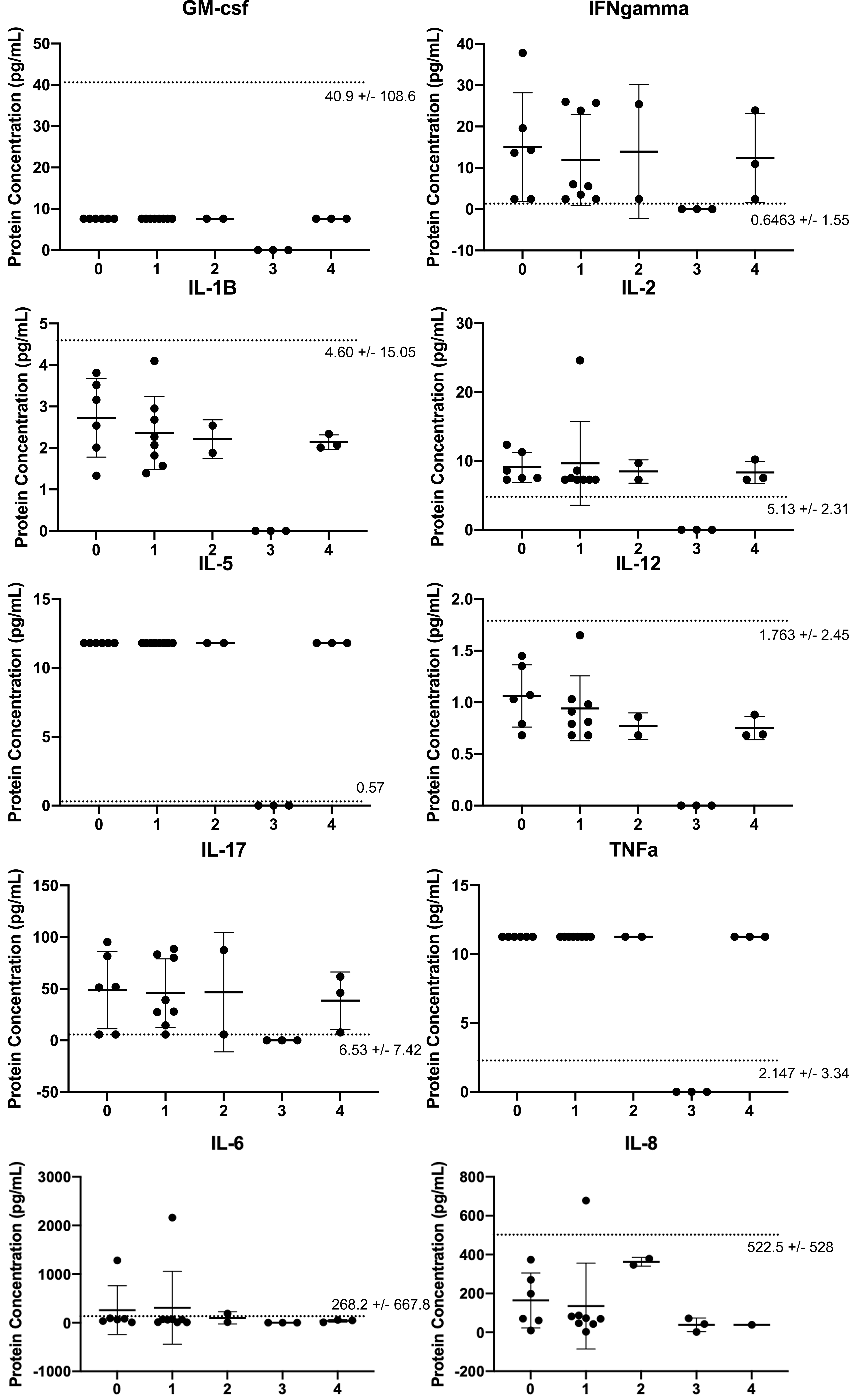

Supplement: Supplementary file 4 — Additional file 4: Figure S4. Protein concentration values of select pro-inflammatory cytokines in terms of past revisions: obstructed cases. Mean with standard deviation is shown in error bars. [file 12987_2021_237_MOESM4_ESM.png]

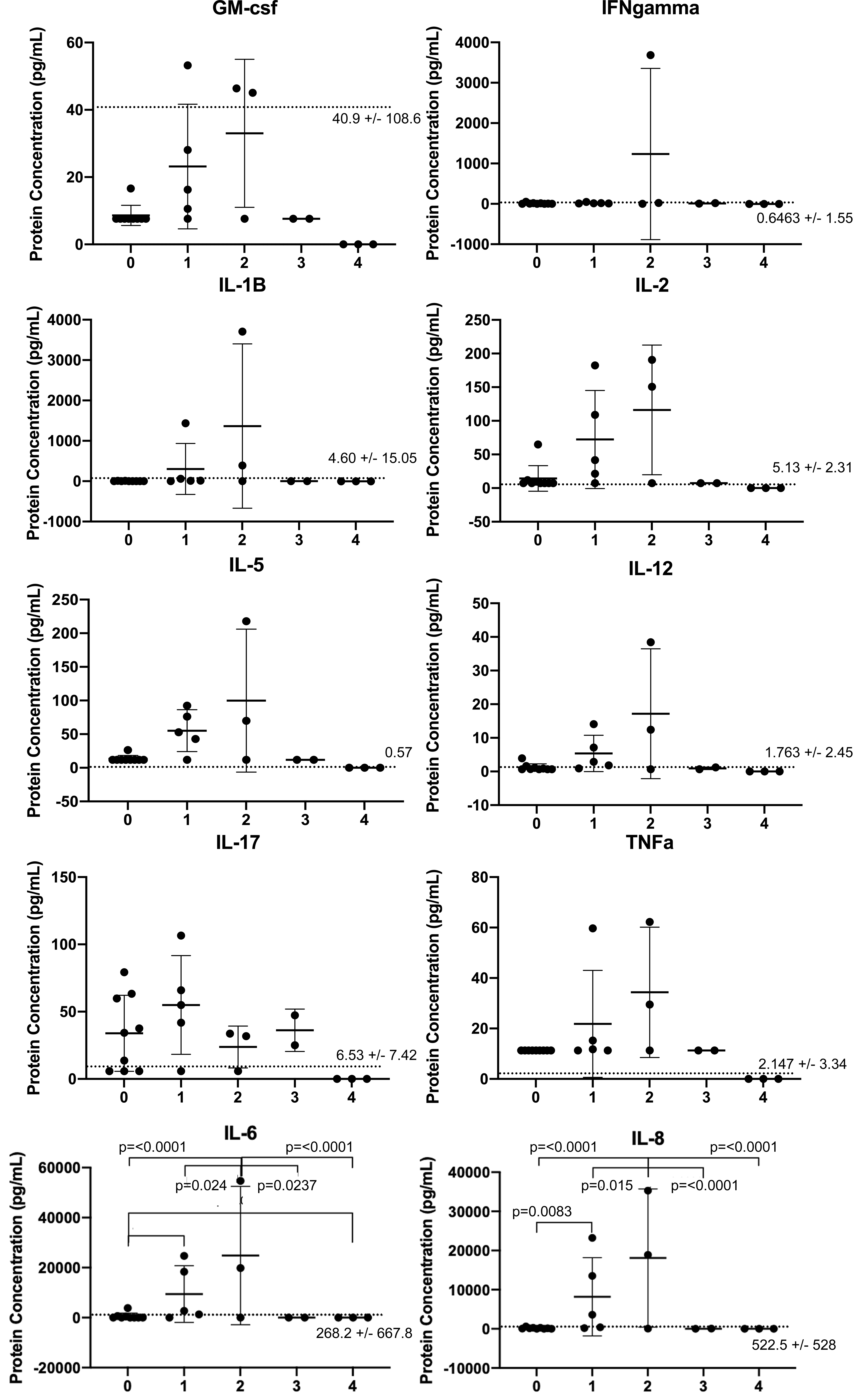

Supplement: Supplementary file 5 — Additional file 5: Figure S5. Protein concentration values of select pro-inflammatory cytokines in terms of past revisions: non-obstructed cases. Mean with standard deviation is shown in error bars. [file 12987_2021_237_MOESM5_ESM.png]

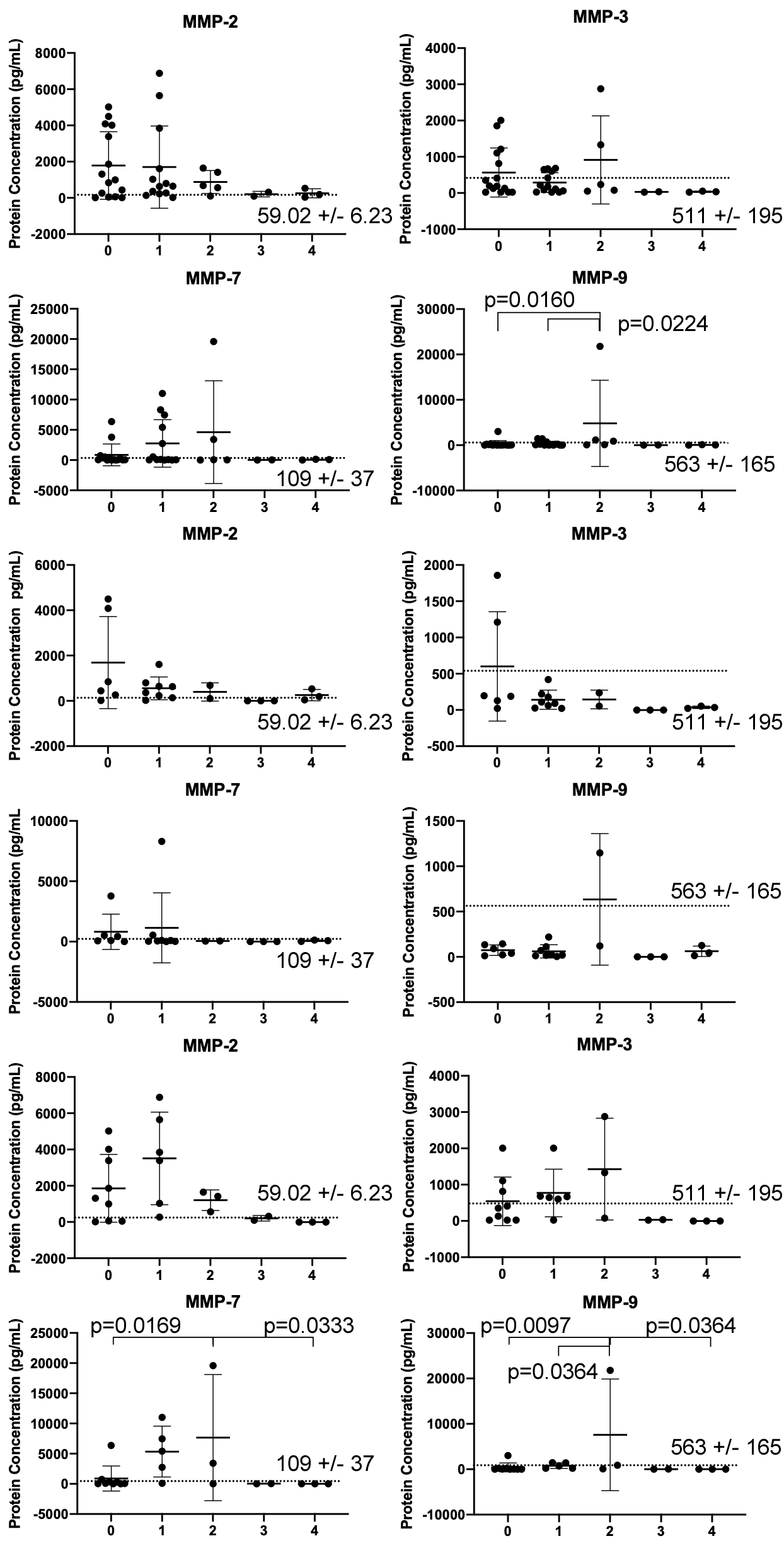

Supplement: Supplementary file 6 — Additional file 6: Figure S6. Protein concentration values of select MMPs (Matrix Metalloproteinase) in terms of past revisions: (A) unparsed data, then (B) obstructed vs. (C) non-obstructed cases. Mean with standard deviation is shown in error bars. [file 12987_2021_237_MOESM6_ESM.png]
